# Scalable Synthesis of Ag Networks with Optimized Sub-monolayer Au-Pd Nanoparticle Covering for Highly Enhanced SERS Detection and Catalysis

**DOI:** 10.1038/srep37092

**Published:** 2016-11-15

**Authors:** Tianyu Li, Sascha Vongehr, Shaochun Tang, Yuming Dai, Xiao Huang, Xiangkang Meng

**Affiliations:** 1 National Laboratory of Solid State Microstructures, Collaborative Innovation Center of Advanced Microstructures, College of Engineering and Applied Sciences, and Institute of Materials Engineering, Nanjing University, Jiangsu, P. R. China; 2 School of Materials Engineering, Nanjing Institute of Technology, Jiangsu, P. R. China

## Abstract

Highly porous tri-metallic Ag_*x*_Au_*y*_Pd_*z*_ networks with a sub-monolayer bimetallic Au-Pd nanoparticle coating were synthesized via a designed galvanic replacement reaction of Ag nanosponges suspended in mixed solutions of HAuCl_4_ and K_2_PdCl_4_. The resulting networks’ ligaments have a rough surface with bimetallic nanoparticles and nanopores due to removal of Ag. The surface morphology and composition are adjustable by the temperature and mixed solutions’ concentration. Very low combined Au and Pd atomic percentage (1−*x*) where *x* is atomic percentage of Ag leads to sub-monolayer nanoparticle coverings allowing a large number of active boundaries, nanopores, and metal-metal interfaces to be accessible. Optimization of the Au/Pd atomic ratio *y*/*z* obtains large surface-enhanced Raman scattering detection sensitivity (at *y*/z = 5.06) and a higher catalytic activity (at *y*/*z* = 3.55) toward reduction reactions as benchmarked with 4-nitrophenol than for most bimetallic catalysts. Subsequent optimization of *x* (at fixed *y*/*z*) further increases the catalytic activity to obtain a superior tri-metallic catalyst, which is mainly attributed to the synergy of several aspects including the large porosity, increased surface roughness, accessible interfaces, and hydrogen absorption capacity of nanosized Pd. This work provides a new concept for scalable synthesis and performance optimization of tri-metallic nanostructures.

Nanostructures of noble metals such as gold (Au), silver (Ag), platinum (Pt), and palladium (Pd) have received extensive attention due to their promising applications in catalysis^1^,^2^,^3^, biosensors^4^,^5^, optical devices^6^,^7^, and surface-enhanced Raman scattering (SERS) detection^8^,^9^. Their properties for these applications depend heavily on surface morphology^10^,^11^ and sizes^12^,^13^,^14^, including for example hierarchically distributed pores’ sizes^15^,^16^ that provide superior access to active sites. Designed synthesis of nanostructures with novel surface morphologies and tailored sizes has therefore become very important. Although nanoparticles (NPs) show high catalytic activities due to high ratio of surface atoms and size effect^17^,^18^, they easily aggregate^19^ and repeated use is difficult. Bulk materials with network-like nanostructures can be very easily collected after application and do not suffer from aggregation, consistently keeping their specific local nanostructure and high specific surface area^20^. In particular, such structures can also provide further SERS hotspots along their third dimension^3^,^21^, which results in a high detection sensitivity^22^.

Multi-metallic structures provide many active inter-metallic interfaces where electronic structure is changed^22^,^23^. Many investigations focused on Ag-Au bimetallic structures due to their relatively low-cost and high performance, for example Ag-Au core-shell NPs^24^, dendrites^25^, alloy NPs^26^ and alloy/graphene hybrids^27^ with excellent catalytic activities. Bi-metallic compounds already allow tuning the catalytic activity via composition ratios. As a comparison, tri-metallic compounds can provide far more opportunities for the tailoring of unique shapes and thus multi-parameter optimization for more fine property tuning^28^,^29^. In particular, tri-metallic interfaces are more active due to the presence of crystal defects and fast electron interchange. So far, still little effort has focused on tri-metallic networks, which promise large enhancements in performance (specifically for the targeted catalysis and SERS detection) due to the synergistic combination of the advantages of networks and multi-metallicity.

Catalysis and SERS detection depend both highly on surface characteristics, and focusing only on the surfaces of compounds allows very low contents of expensive noble metals. Sub-monolayer coverings are superior over core-shell structures with complete coatings^30^. Reduction of metal ions on the surface of a sacrificial metal, better known as galvanic replacement reaction (GRR), is expected to be a simple yet versatile method to achieve such. The products from GRR are more stable than those synthesized by loading NPs onto surfaces^31^,^32^. Due to the simultaneous consumption of substrate material while adding newly growing crystals, GRR can result in hierarchically porous surfaces with many active corners and edges^33^. However, previous reports mainly focused on the synthesis of hollow^34^,^35^, core-shell^24^,^25^,^36^, or alloyed^37^,^38^ nanostructures via GRR, accessible active inter-metal interfaces are limited, and the necessary amount of very expensive metals like Ru^34^, Pd^35^,^38^, Pt^37^ and Au^36^ are high. It is expected that products through a GRR starting with highly porous interconnecting networks should have hierarchical structures with pore sizes from micron to only a few nanometers^39^. Therefore, developing a general, scalable and low-cost strategy to prepare such tri-metallic structures is of great significance.

Herein, we report the rational design and fabrication of highly porous trimetallic Au-Pd-Ag networks with sub-monolayer Au-Pd nanoparticle coating via a GRR in mixed solutions of chlorauric acid (HAuCl_4_) and potassium chloropalladite (K_2_PdCl_4_). A temperature of 60 °C allows the formation of bimetallic NPs’ coating, avoiding larger grown structures such as nanoplates and irregular aggregates at lower and higher temperatures, respectively. Adding too few Au and Pd NPs can only provide bimetallic Ag/Au and Ag/Pd interfaces, not tri-metallic interfaces. With too many NPs, only bimetallic Au/Pd interfaces are accessible and none of the nanopores due to removed Ag. The concentrations were therefore initially adjusted so that the NPs surface coverage *σ* would likely be only about half a monolayer (*σ* ~ 0.5). The resulting Ag_*x*_Au_*y*_Pd_*z*_ compound has therefore *x* equal to about still 90%. The suggested process was confirmed by optimizing the Au/Pd atomic ratio *y*/*z* to obtain large surface-enhanced Raman scattering detection sensitivity. Also, we pre-optimized the catalytic activity first by adjusting the Au/Pd atomic ratio. However, the catalytic activity also depends strongly on the surface coverage σ, therefore *x* was then optimized while holding the Au/Pd ratio fixed. This near optimization in the 2D parameter space leads to a superior catalytic activity (as benchmarked with 4-NP reduction) over most reported bimetallic catalysts.

## Results and Discussion

**Figure 1:**
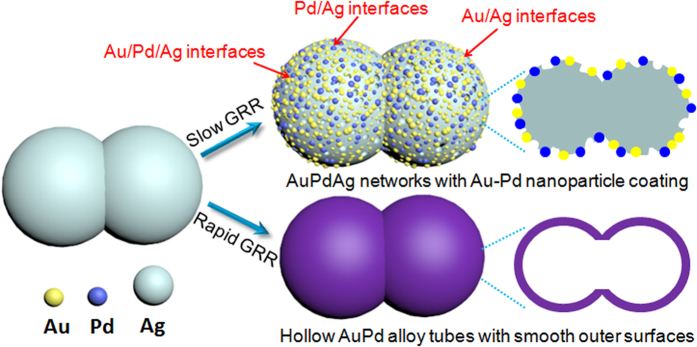
Illustration of the formation of AgAuPd networks with a sub-monolayer Au-Pd nanoparticle coating allowing different types of inter-metallic interfaces via a GRR of Ag sponges in mixed solutions. Note that the connected two spheres schematically represent Ag beads in one ligament of the nanosponges.

Details of the synthesis as well as the related calculations are described in the Supporting Information. Figure 1a illustrates the formation of AgAuPd networks with a sub-monolayer Au-Pd nanoparticle covering via a GRR of Ag sponges in mixed solutions containing HAuCl_4_ and K_2_PdCl_4_. On one hand, the specific nanostructures of the covering are determined by the mixed solutions’ concentration. A rough surface with isolated NPs is the product from the designed very low concentrations due to a small amount of removed Ag while a hollow tube with a smooth surface is obtained instead when the concentration is relatively high. The former is desirable for SERS detection and catalysis due to its increased surface roughness and accessible inter-metal interfaces. On the other hand, the composition of the covering is dependent on the GRR rate. Generation of isolated Au and Pd NPs (not AuPd alloys with uniformly mixed Pd and Au atoms inside the NPs) is due to the separate reduction of precursor ions and their subsequent selective growth at slow GRR rates. During a slow GRR process, well-dispersed nuclei form firstly on the surface starting from a selective replacement of the Ag ligaments at point defects. The subsequent selective growth of these nuclei leads to larger particles. This is different the generation of alloys from the fast one-step reductions of mixed metal ions with a strong reducer^26^ or microwave-assisted fast and selective heating^27^, and a GRR with an assistance of a strong reducer^38^. It is a very interesting and somewhat surprising result. Indeed, since the GRR took place from the surface of the Ag ligaments, the resultant product was expected to be either alloy or isolated Au and Pd NPs. In our GRR process, combined very low concentrations of mixed metal ions and a low reaction temperature allows the formation of the latter that has different types of inter-metallic interfaces (Ag-Pd, Au-Ag, and Ag-Au-Pd) on the Ag-ligaments.

The GRR proceeds according to the following equations:


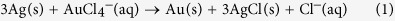



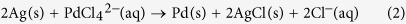


The oxidation of Ag^0^ into Ag^+^ leads to the removal of Ag. At the same time, Au^0^ and Pd^0^ are deposited, but not necessarily at the location where Ag is removed. The difference between the standard reduction potential of the AuCl_4_^−^/Au redox pair (0.99 V vs standard hydrogen electrode (SHE)) and the PdCl_4_^2−^/Pd redox pair [0.83 V vs SHE] and the mild reaction conditions (including low temperature and concentrations) result in a separate deposition of Pd^0^ and Au^0^, but not simultaneous reduction.

In order to obtain dispersed NPs, the GRR cannot be too fast or too slow. The reaction rate depends on the concentrations and reaction temperature. At room temperature, nanosheets obtain on the ligaments (Figure S1a, Supporting Information), while above 65 °C, large aggregations form (Figure S1b, Supporting Information). In order to maximize the density of accessible active sites via deposition of NPs by GRR, we chose 60 °C for the GRR synthesis. When *C*_Au_ and *C*_Pd_ are ten times the typical value (Figure S1c,d, Supporting Information), there is no accessible Ag left, and the ligaments are hollow AuPd alloy tubes with smooth outer surfaces (see the scheme in Fig. 1). As discussed in the introduction, too low as well as too high concentrations provide only bimetallic interfaces. The number of tri-metallic interfaces should be maximized near a 50% surface coverage *σ* ≅ 3/4 × (*R*/*r*) × *s*, where *R* is the network ligament radius and *s* the ratio of the number of added atoms divided by the number of initial Ag atoms. The concentrations were therefore chosen so that the resulting Ag_*x*_Au_*y*_Pd_*z*_ compound will have *x* ~ 0.9 (see details in “Theoretical calculations”).

**Figure 2:**
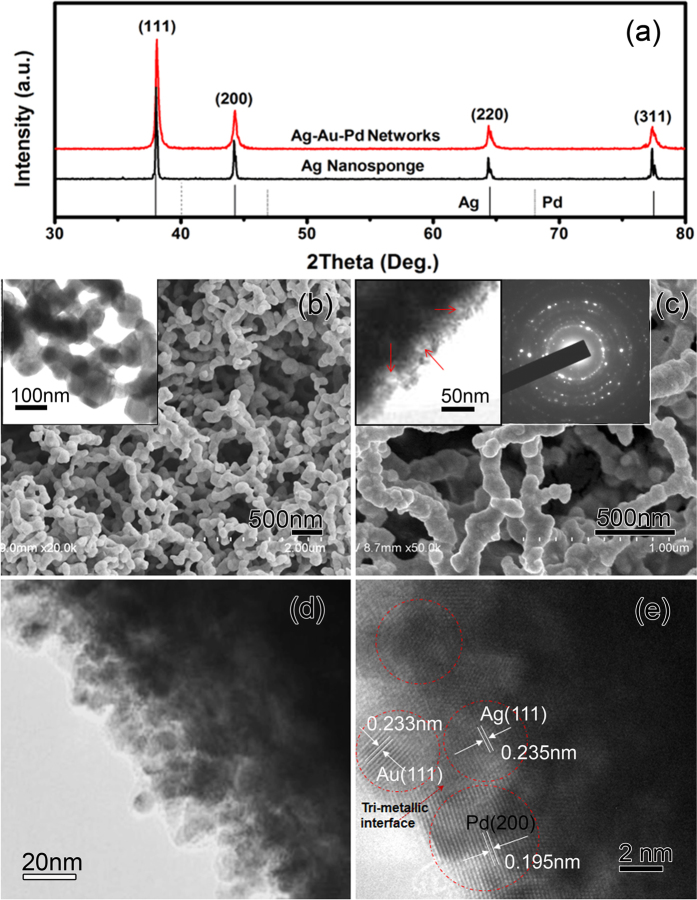
(**a**) XRD patterns of the initial Ag sponges and typical Ag-Au-Pd networks (the characteristic peaks of bulk Ag and Pd are provided for comparison). FESEM images showing the Ag sponge (**b**) before and (**c**) after GRR; insets show corresponding TEM images and SAED pattern; (**d**) local magnification of the ligament’s edge, and (**e**) HRTEM image of a tri-metallic interface.

Figure 2a shows XRD patterns of the initial Ag sponges and the typically resulting Ag-Au-Pd products. The peaks can all be ascribed to the diffractions from (111), (200), (220) and (311) of face-centered cubic Ag (JCPDF No. 04–0783). Only a slight shift and widening of the peaks due to the GRR are observed. The intensity ratio between the (200) and (111) diffraction peaks is 0.42 for Ag sponges, which is lower than the 0.53 of bulk Ag, because the deposition of Ag atoms tends to happen at the facet (111) rather than other high-index facets. The ratio 0.35 for the Ag-Au-Pd networks being yet lower indicates that also the deposition of Au and Pd atoms occurs predominantly at low-index facets. The Ag precursor’s network links, also called ligaments, appear necklace-like (see the FESEM image in Fig. 2b). The surface of the ligaments is smooth (see TEM image in the inset), and the ligament diameter as measured across the necklace beads is about 80–110 nm.

Figure 2c shows a SEM image of the typical product. The deposited NPs can only be seen clearly in TEM images (left inset), where also the pores between the deposited NPs become visible (see red arrows). The surface roughness could be ascribed to the poor epitaxial growth of Pd on Ag as caused by the relatively large difference (~4.8%) in lattice constant between Ag and Pd. The SAED pattern (right inset) recorded from a single ligament confirms the expected polycrystalline nature. The continuous diffraction rings are due to the newly formed NPs, while the bright spots are due to the much larger, single crystalline Ag ligament. The locally magnified TEM image of the ligament’s edge (Fig. 2d) indicates that the NPs have an average diameter of about 5 nm. The HRTEM analysis (Fig. 2e) shows that the Au and Pd particles often touch on the Ag surface, resulting in the desired Ag-Au-Pd tri-metallic interfaces. Various crystallographic orientations are identified, and the observable lattice spacing in the dashed circles are assigned to Ag (111), Au (111), and Pd (200) planes, respectively. The average diameter of the patches is consistent with that of NPs, which implies that Au and Pd are isolated single-crystalline NPs.

**Figure 3:**
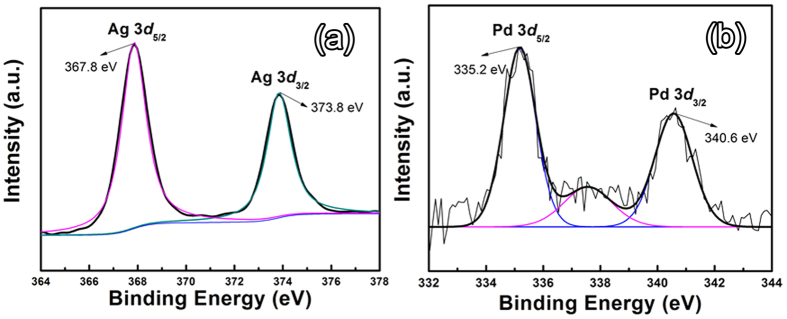
High resolution XPS spectra of (**a**) Ag3*d* and (**b**) Pd3*d* peaks recorded from the typical Ag-Au-Pd sample.

XPS is a highly sensitive analysis technique to study the elemental compositions. Figure 3 shows the characteristic XPS spectra of Ag3*d* and Pd3*d* recorded from the AgAuPd networks. Two well-resolved strong peaks at binding energies of 367.8 and 373.8 eV (Fig. 2a) can be attributed to Ag 3*d*_5/2_ and 3*d*_3/2_, which are typical for Ag(0). The peaks at the binding energies of 335.2 and 340.6 eV with a spin-orbit separation of 5.40 eV (Fig. 2b) can be assigned to the Pd3*d*_5/2_ and 3d_3/2_, respectively. Simultaneous presence of the two peaks confirms the formation of metallic Pd(0). Therefore, on the basis of the XPS, XRD analysis as well as TEM and HRTEM results, the deposited Pd and Au stay on the surface of the Ag ligaments as separated NPs rather than forming an alloy layer.

**Figure 4:**
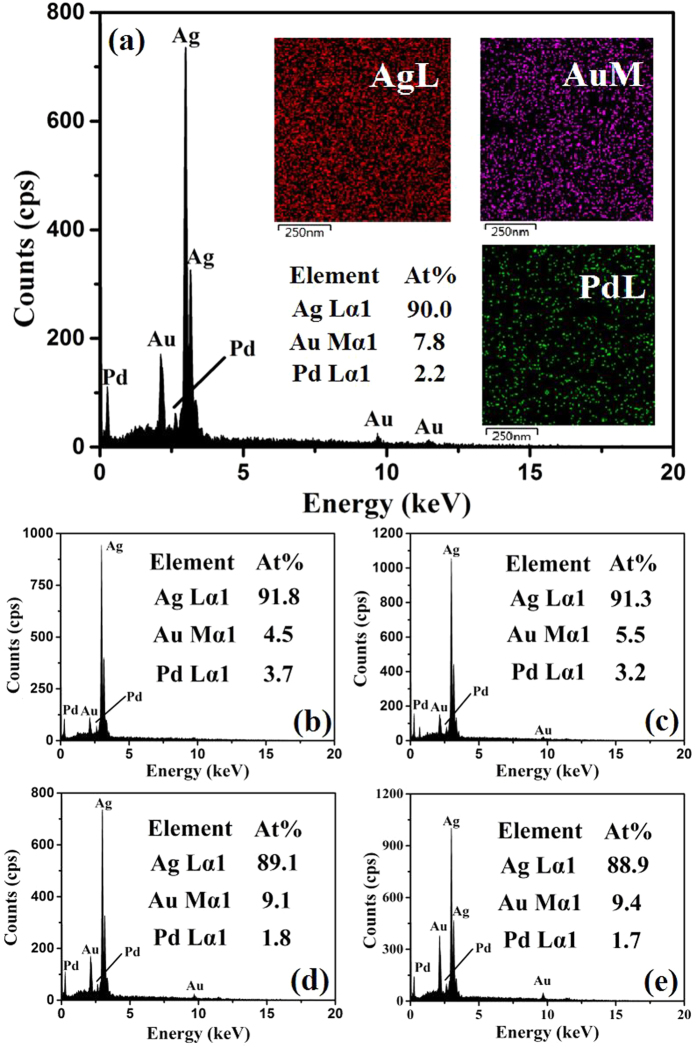
(**a**) EDX patterns of the typical Ag_0.90_Au_0.08_Pd_0.02_ networks, (**b**) Ag_0.92_Au_0.04_Pd_0.04_, (**c**) Ag_0.91_Au_0.06_Pd_0.03_, (**d**) Ag_0.89_Au_0.09_Pd_0.02_, and (**e**) Ag_0.89_Au_0.095_Pd_0.015_. Insets of (**a**) are X-ray elemental mappings of elemental Ag, Au, and Pd recorded from the typical Ag_0.90_Au_0.08_Pd_0.02_.

The typical EDS spectrum (Fig. 4a) confirms low atomic percentages of Au and Pd in the products. The corresponding X-ray mappings (the inset) reveal that the Au and Pd are distributed homogeneously on the micro scale. Semi-quantitative EDS analysis estimates the Au and Pd atomic percentages to be 7.8 and 2.2 percent, respectively. In addition, the atomic percentage of Au and Pd within the XPS penetration depth of <5 nm is estimated to be 7.8 ± 0.3% and 2.3 ± 0.2%, which further confirms a rather thin layer (sub-monolayer) of Au-Pd nanoparticle deposition. These values are roughly consistent with the ICP-AES results indicating that it was composed of Ag, Au and Pd with 89.6 and 7.6 and 2.8 in atomic percentage, respectively. We have furthermore been checked by titration of the Ag^+^ ions in the reaction solution after separating the product. They can therefore be taken as quite accurate. Thus, the typical sample is denoted as Ag_0.90_Au_0.078_Pd_0.022_. Some main products obtained with different concentrations are listed in Table S1, Supporting Information, where the Au/Pd atomic ratios (*y*/*z*) are also given. Thus, the composition is adjustable by the used concentrations.

**Figure 5:**
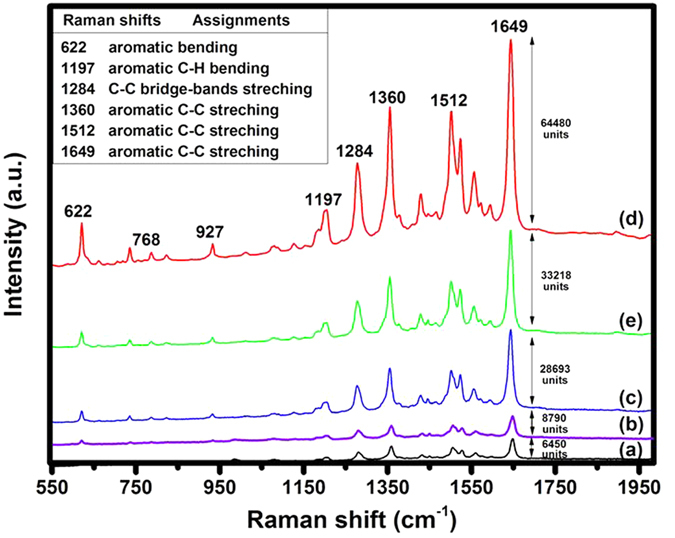
Raman spectra of 10^−6^ M RhB on different substrates, first on precursor Ag sponge (**a**) and then on the different resulting networks of (**b**) Ag_0.918_Au_0.045_Pd_0.037_, (**c**) Ag_0.90_Au_0.078_Pd_0.022_, (**d**) Ag_0.891_Au_0.091_Pd_0.018_, and (**e**) Ag_0.889_Au_0.094_Pd_0.017_.

Figure 5 shows Raman spectra of Rhodamine B (10^−6^ M) adsorbed on networks with different Au/Pd atomic ratios. Comparing with Ag sponges, all the Ag-Au-Pd products show pronounced enhancements. The Ag_0.891_Au_0.091_Pd_0.018_ network (*y*/*z* = 5.06) shows the highest signals; the intensity is nearly tenfold enhanced over the Ag sponge precursor. Since SERS detection is highly dependent on surface characteristics, this significant SERS enhancement originates predominantly from their 3D porous structures^40^ and the surface roughening effect (see Fig. 2b,d). That is to say, it originates from that the transformed surface provides more possibilities for molecules to deposit especially into the boundaries and crevices between Ag (and its newly formed pores) and the grown Au NPs, as such geometries enhance the electromagnetic field around the molecules due to their Plasmon resonances in the visible region^41^. Silver has the highest detection sensitivity among the three metals. With gradual increase of the combined atomic percentages of Au and Pd (from Fig. 5a,d), SERS intensity of signals increases obviously, which is mainly due to a strong increase in surface roughness of the active Ag substrate due to the formation of many nanopores, NPs and inter-metal interfaces. When more and more SERS-inactive Au and Pd were deposited, the surface roughness was lowered, resulting in an obvious decrease of the intensity (from d to e). This may be simply due to the fact that deposited Au and Pd increasingly starts to change and even hide the rough SERS-active Ag structure beneath. In addition, the Au/Pd atomic ratio also increases, much more Au than Pd was induced in the all Ag-Au-Pd samples, which leads to that the deposited Au covered the active bimetallic and trimetallic interfaces.

The morphology and compositional information obtained motivated us to explore their catalytic activity. The reduction of 4-NP by NaBH_4_ is a model reaction and easily monitored by UV-visible absorption spectroscopy^42^. The total reaction of 4-NP reduction by NaBH_4_ in the presence of catalysts is the following equation:


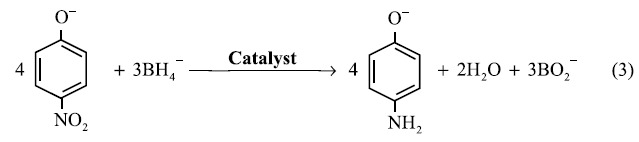


**Figure 6:**
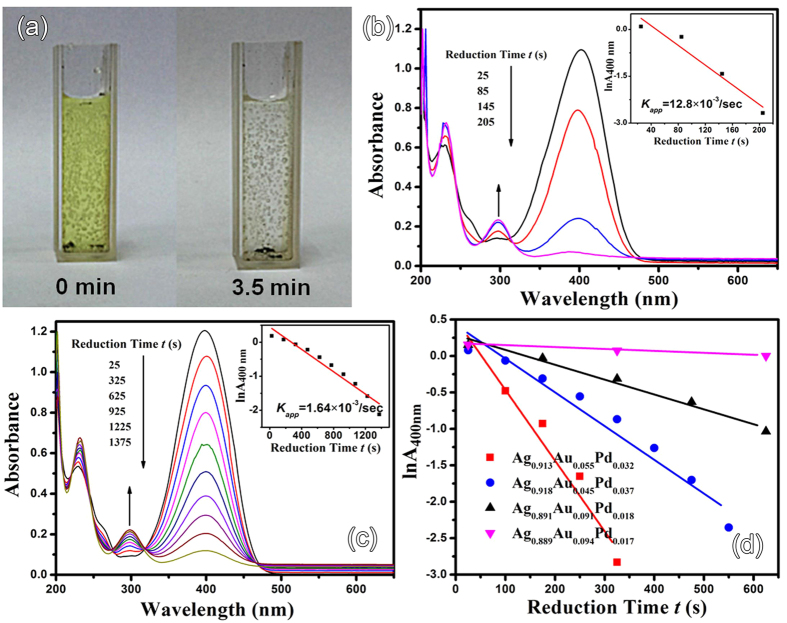
Photographs showing the color change of the 4-NP solution after reaction for 3.5 min under addition of Ag_0.90_Au_0.078_Pd_0.022_ networks (**a**) UV-Vis absorption spectra of 4-NP during reduction by NaBH_4_ in the presence of 0.5 mg of (**b**) Ag_0.90_Au_0.078_Pd_0.022_ and (**c**) precursor Ag sponge. The inset shows the logarithm of the absorbance at 400 nm vs. reduction time *t*. (**d**) Plots of lnA_400nm_ versus *t* for different Ag-Au-Pd networks.

Without the catalyst, the reduction will not proceed, because the kinetic barrier between nitrophenolate anions and BH_4_^−^ is too high. However, when even only 0.5 mg of dry Ag-Au-Pd sponges were added, the reduction of 4-NP proceeds rapidly as can be seen from the bleaching of the yellow color (Fig. 6a). Due to the highly porous nature of the Ag sponge, the volume seems to be larger than that for 0.5 mg of solid Ag. A linear decay of lnA_400_ over reduction time *t* was obtained (insets of Figs 6b and 4c). The calculated reduction rate, also called apparent rate constant *K*_app_, is 12.8 × 10^−3^/s for Ag_0.90_Au_0.078_Pd_0.022_. The catalytic activity increased nearly eight times in comparison with the pure Ag nanosponges although the latter also has a porous nanostructure. Thus, the deposition of Au-Pd NPs to Ag ligaments resulting in a significant activity enhancement should be originated from their synergistic interactions. The plots of lnA_400nm_ for other samples are shown in Fig. 6d. The catalyst can be reused and has not been found to decrease significantly in activity.

**Figure 7:**
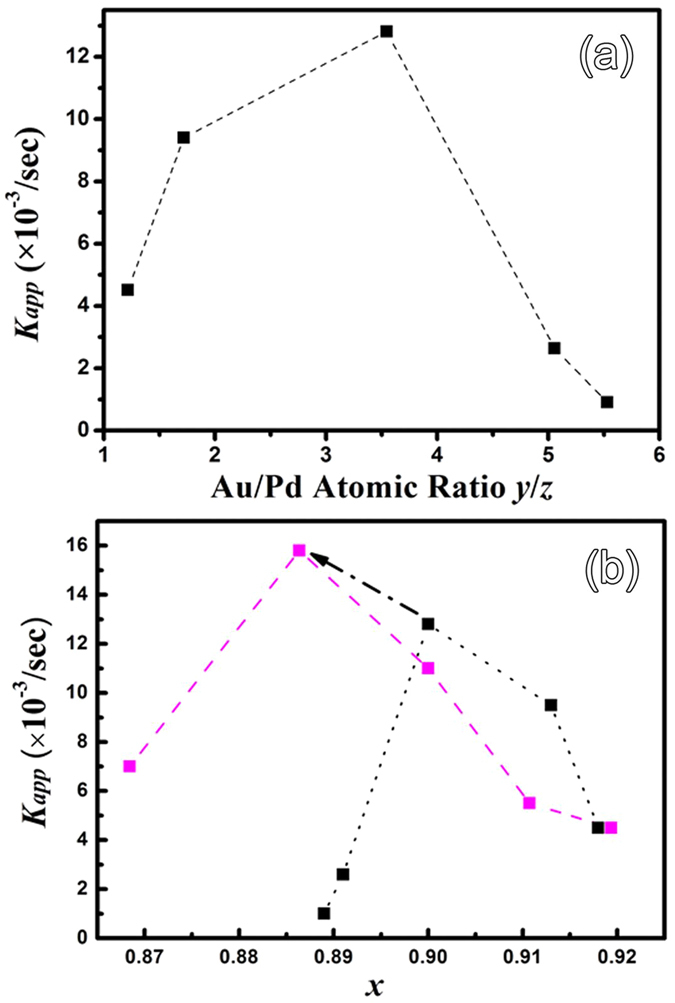
(**a**) Apparent rate constant *K*_app_ vs. Au/Pd atomic ratio *y/z*. (**b**) Second optimization step, showing *K*_app_ vs. *x*. The black curve is the very same as in (**a**) again, but now presented in *x*-space (seen from a different angle). The new pink curve has a fixed *y/z* and cannot be seen in (**a**), because it is orthogonal to the direction *y/z*. In *x* versus *y/z* space, the new curve crosses the previous one almost at an right angle, which allows to reach a high new constrained maximum when departing from the previous one (see the black arrow).

The largest rate constant obtains at an Au/Pd atomic ratio of *y*/*z* = 3.55 (Fig. 7a). It is known that Pd is far more catalytic than Au or Ag in the reduction of 4-NP, especially small Pd NPs are highly catalytic^43^. Studies show that nanosized Pd catalyst allows a new reaction pathway of 4-NP reduction that is not available at all in the absence of Pd, which will be discussed in detail later. Thus one usually expects a steep rise in catalytic activity when inducing the catalytic active Pd. It is remarkable that the catalytic activity here also rises steeply, but from a 1:1 ratio of Au versus Pd. This also indicates that one cannot simply claim that *y*/*z* = 3.55 is most important for optimal catalytic performance, because the parameter *x* has not been held constant and changes in a range from 89% to 92% (blue curve in Fig. 7b). According to σ ≅ ¾(*R*/*r*) *s* and *x* = (1−3*s*)/(1−2*s*), small differences in the amount of Ag translate into large differences in the surface coverage σ. With NPs of *r* = 4 nm radius, *x* = 0.89 has 17% more of its surface covered than *x* = 0.92. Both parameters, *x* and *y*/*z*, strongly influence the number of tri-metallic interfaces, and *x* moreover influences especially the number of accessible Ag nanopores due to removed Ag. We therefore fixed the Au/Pd ratio to the pre-optimized value of *y*/*z* = 3.55 in the mixed GRR solutions. Using 5 mg of Ag precursor led to *x* = 0.9. Therefore, using the same volumes of that GRR solution again, but now with different initial weights of Ag, ensures that *y*/*z* stays constant while *x* changes. The results are shown in the pink curve in Fig. 5b. Note that using 5.0 mg of Ag again leads to almost the same result as before, namely the previous maximum in Fig. 7a (and Fig. 7b on the black curve), illustrating reproducibility. Next, one can immediately see that most of the other new values are much lower than the previous maximum, although they have the pre-optimized *y/z* ratio, proving that *x* and *y*/*z* are indeed equally important. The new maximum is at *x* = 0.89, corresponding to σ ~ 0.76. The new maximum of 15.8 × 10^−3^/s on the pink curve is higher than the previous one on the black curve. This confirms that simply optimizing in a higher dimensional parameter space is extremely likely to lead to better values^44^, especially if departing almost orthogonal to the already performed optimization (see the arrow in Fig. 5b). The result here is still constrained to *y*/*z* = 3.55. A proper walk-in optimization in the full 2D parameter space would result in a slightly different *y/z* and an even better catalytic rate constant. For now, with our two step optimization, the result is that *x* = 0.89 and *y*/*z* = 3.55 are close to optimal for catalysis. Note that *x* = 0.89 was also the best for SERS enhancement. It is found that the discussion of the influence of morphology on SERS and the discussion afterward about the catalytic activity are consistent. The SERS detection and the catalytic activity are both maximal with Ag_*x*_Au_*y*_Pd_*z*_ having *x* at about 0.89.

The ratio of the apparent rate constant *K*_app_ over the total weight of catalyst used is the weight specific activity *k* = *K*_app_/*m*, which facilitates the comparison with the literature due to the use of different amounts of catalysts (see Table 1)^2^,^45^,^46^,^47^,^48^,^49^,^50^,^51^. The catalytic activity of our optimized tri-metallic networks is enhanced over bimetallic catalysts^42^,^45^,^46^,^47^,^52^,^53^,^54^ as expected. It is also however enhanced over other tri-metallic nanostructures like Au bipyramids@AgPd nanodendrites^48^. The catalytic activity increases with the availability of metal-metal interfaces and active surfaces^55^. The significant activity enhancements mainly originate from the synergistic interactions between Au-Pd, metal-metal interfaces, and the geometric factors. Since Pd and Au are far more catalytic than Ag for the 4-NP reduction reactions, the introduction of Pd and Au surfaces leads to an enhancement in the catalytic activity.

Donors like the BH_4_^−^ ions supply electrons to the catalyst and allow the 4-NP on the catalyst to take electrons. The higher catalytic activity of Ag-Au-Pd may be also related to the number of metal-metal interfaces. Ag has a lower work function than Au and Pd. Therefore, electrons near Au/Ag or Au/Pd interfaces transfer to Au and Pd, which become electron enriched regions (“hotspots”)^47^. The more interfaces there are, the more such regions with surplus electrons exist. The regions facilitate the uptake of electrons by 4-NP molecules, while the depleted regions facilitate the BH_4_^−^ donors’ delivering of electrons to the catalyst. As for the influence of the geometric factors, the galvanic replacement process results in a large increase in the surface roughness of the Ag ligaments (see Fig. 2).

Another well-known acceleration of hydrogen involving reactions is due especially to the metallic Pd^47^ acting as a “hydrogen relay system”^56^. The reaction mainly involves an addition of protons to the 4-NP and simultaneous removal of its oxygen^50^. For the production of hydrogen radicals from NaBH_4_:


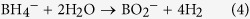


The addition of protons to the 4-NP and the simultaneous removal of its oxygen are described as the following equation:


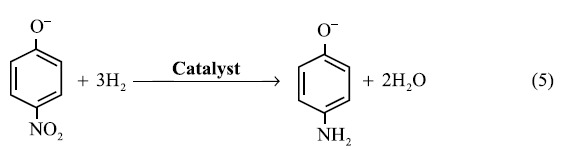


The split of the total reaction (eq. 3) into the hydrogen producing part (eq. 4) and a hydrogen consuming reaction (eq. 5) illustrates the great advantage of a hydrogen absorbing material that insures that these reactions can proceed almost independently. Because of the hydrogen storage capacity, Pd facilitates the intermediate storage of hydrogen radicals that can react with 4-NP, while the catalyst also activates the nitro group^51^. BH_4_^−^ and 4-NP need neither be absorbed close to each other nor simultaneously. The catalysts are not just effective electron relay systems but also hydrogen relay systems^56^. The metal-metal interfaces greatly enhance electron transfer, while the Pd enhances the hydrogen transfer. These two aspects, namely metal-metal interfaces and Pd, explain the catalytic ability of *bimetallic* Ag-Pd nanostructures. The much further improved catalytic activity of *tri*-metallic structures such as our Ag-Au-Pd networks derives from that the available configurations of metal-metal interfaces, pores and suchlike, are much more diverse. This can allow new reaction pathways not otherwise available. Moreover, these more diverse structures can be optimized inside a parameter space with more dimensions, such as different particle sizes for Au, Pd NPs and locations and so on. This allows a much better optimization^44^. Our optimization here was only performed partially and only in a two dimensional parameter space (*x* and *y/z*), but the results already confirm this general understanding.

## Conclusions

In summary, trimetallic Ag_x_Au_y_Pd_z_ network-like nanostructures were synthesized via a designed GRR process. The original Ag sponge develops pores, while the growing Au and Pd NPs cover the network ligaments’ surfaces. The surface coverage is less than a mono-layer, namely, assuming no stacking of deposited NPs via GRR, only about a three quarter surface coverage after optimizing the catalytic activity in the two dimensional (*x* × *y*/*z*) parameter space. Large specific surface area, bi- and tri-metallic interfaces, availability of nanopores, and the hydrogen storage capacity of Pd endow the tri-metallic networks with high catalytic activity. The expensive metals Au and Pd are only at the accessible surface, on top of an inexpensive substrate. The enhanced porosity results in a high sensitivity for SERS. Catalytic activity and SERS detection are both maximal with Ag_*x*_Au_*y*_Pd_*z*_ having *x* at about 0.89. Because of their convenient preparation, large-scale production and high performance, such trimetallic AgAuPd nanostructures will find promising potential applications in SERS detection and catalysis. Much further research will focused on full distinguishing how the very low surface contents and roughening contribute to SERS and catalytic activities in the 3D parameter space.

## Additional Information

**How to cite this article**: Li, T. *et al*. Scalable Synthesis of Ag Networks with Optimized Sub-monolayer Au-Pd Nanoparticle Covering for Highly Enhanced SERS Detection and Catalysis. *Sci. Rep.* **6**, 37092; doi: 10.1038/srep37092 (2016).

**Publisher’s note:** Springer Nature remains neutral with regard to jurisdictional claims in published maps and institutional affiliations.

## Supplementary Material

Supplementary Information

## Figures and Tables

**Table 1:** Comparison of apparent rate constants (*K*_app_) and activity factor (*k*) of different catalysts for the reduction of 4-NP.

Catalysts	Quantity/mg	Rate constant *K*_app_/(10^−3^ s^−1^)	Activity factor (*k*)/ (10^−3^ s^−1^ mg^−1^)	Refs
Ag_0.90_Au_0.078_Pd_0.022_	0.5	15.80	31.60	This work
Au@AgPd NDs	/	6.85	/	48
Ag/Au NDs	0.6	6.07	10.11	46
Ag/Pd NPs	1.0	8.3	8.3	47
Ag/Pt NWs	1.0	6.93	6.93	49
Au/Ni NSs	8.0	1.27	0.16	45
Au-Pd/carbon	1.0	14.6	14.6	50
Fe_3_O_4_@C@Ag-Au	0.6	15.8	26.3	51
Ag sponges	0.5	1.64	3.28	This work
Au NPs	10	13.85	1.385	2

NDs: nanodendrites, NWs: nanowires, NSs: nanostructures, NPs: nanoparticles.

## References

[b1] Nursanto E. B. Gold catalyst reactivity for CO_2_ electro-reduction: From nano particle to layer. Catal. Today 260, 107–111 (2016).

[b2] Tang M. Y., Huang G. B., Li X. X., Pang X. B. & Qiu H. X. A facile approach to fabricate Au nanoparticles loaded SiO_2_ microspheres for catalytic reduction of 4-nitrophenol. Mater. Chem. Phys. 162, 31–40 (2015).

[b3] Johnston P., Carthey N. G. & Hutchings J. Discovery, development, and commercialization of gold catalysts for acetylene hydrochlorination. J. Am. Chem. Soc. 137, 14548–14557 (2015). 26529366 10.1021/jacs.5b07752

[b4] Miranda O. R. . Colorimetric bacteria sensing using a supramolecular enzyme-nanoparticle biosensor. J. Am. Chem. Soc. 133, 9650–9653 (2011). 21627131 10.1021/ja2021729 PMC3120917

[b5] Maxwell D. J., Taylor J. R. & Nie S. M. Self-assembled nanoparticle probes for recognition and detection of biomolecules. J. Am. Chem. Soc. 124, 9606–9612 (2002). 12167056 10.1021/ja025814p

[b6] Prieto F. . An integrated optical interferometric nanodevice based on silicon technology for biosensor applications. Nanotechnology 14, 907–912 (2003).

[b7] Joshi G. K. . Ultrasensitive photoreversible molecular sensors of azobenzene functionalized plasmonic nanoantennas. Nano Lett. 14, 532–540 (2014). 24393014 10.1021/nl403576c

[b8] Xie J. P. . The synthesis of SERS-active gold nanoflower tags for *in vivo* applications. ACS Nano 2, 2473–2480 (2008). 19206281 10.1021/nn800442q

[b9] Duy P. K., Yen P. T. H. Chun S., Ha V. T. T. & Chung H. Carbon fiber cloth-supported Au nanodendrites as a rugged surface-enhanced Raman scattering substrate and electrochemical sensing platform. Sensor Actuat. B-Chem. 225, 377–383 (2016).

[b10] Silva A. G. M. . Controlling size, morphology, and surface composition of AgAu nanodendrites in 15s for improved environmental catalysis under low metal loadings. ACS Appl. Mater. Interfaces 7, 25624–25632 (2015). 26544682 10.1021/acsami.5b08725

[b11] Wang Y. X. . Au/Ag bimetal nanogap arrays with tunable morphologies for surface-enhanced raman scattering. RSC Adv. 5, 7454–7460 (2015).

[b12] Murzin D. Y. On cluster size dependent activity and delectivity in heterogeneous catalysis. Catal. Lett. 142, 1279–1285 (2012).

[b13] Fenger R. . Size dependent catalysis with CTAB-stabilized gold nanoparticles. Phys. Chem. Chem. Phys. 14, 9343–9349 (2012). 22549475 10.1039/c2cp40792b

[b14] Hajfathalian M. . Photocatalytic enhancements to the reduction of 4-nitrophenol by resonantly excited triangular gold-copper nanostructures. J. Phys. Chem. C 119, 17308–17315 (2015).

[b15] Tang S. C., Vongehr S., Zheng Z., Ren H. & Meng X. K. Facile and rapid synthesis of spherical porous palladium nanostructures with high catalytic activity for formic acid electro-oxidation. Nanotechnology 23, 255606 (2012). 22652508 10.1088/0957-4484/23/25/255606

[b16] Zhu Z., Sun H., Liu H. & Yang D. PEG-directed hydrothermal synthesis of alumina nanorods with mesoporous structure via AACH nanorod precursors. J. Mater. Sci. 45, 46–50 (2010).

[b17] Semagina N., Renken A. & Minsker L. K. Pd nanoparticle size effect in 1-hexyne selective hydrogenation. J. Phys. Chem. C 111, 13933–13937 (2007).

[b18] Murzin D. Y. Thermodynamic analysis of nanoparticle size effect on catalytic kinetics. Chem. Eng. Sci. 64, 1046–1052 (2009).

[b19] Roca A. G. . Effect of nanoparticle and aggregate size on the relaxometric properties of MR contrast agents based on high quality magnetite nanoparticles. J. Phys. Chem. B 113, 7033–7039 (2009). 19378984 10.1021/jp807820s

[b20] Wang X. Y. . Large-scale fabrication of porous bulk silver thin sheets with tunable porosity for high-performance binder-free supercapacitor electrodes. RSC Adv. 5, 45194–45200 (2015).

[b21] Lee S. . Utilizing 3D SERS active volumes in aligned carbon nanotube scaffold substrates. Adv. Mater. 24, 5261–5266 (2012). 22836924 10.1002/adma.201200645

[b22] Tang S. C. . Versatile synthesis of high surface area multimetallic nanosponges allowing control over nanostructure and alloying for catalysis and SERS detection. J. Mater. Chem. A 2, 3648–3660 (2014).

[b23] Zhu C. Z., Guo S. J. & Dong S. J. Facile fabrication of nanoporous Au-Pd bimetallic foams with high catalytic activity for 2-nitrophenol reduction and SERS property. Chem. Eur. J. 19, 1104–1111 (2013).23180616

[b24] Haldar K. K., Kundu S. & Patra A. Core-size-dependent catalytic properties of bimetallic Au/Ag core-shell nanoparticles. ACS Appl. Mater. Interfaces 6, 21946–21953 (2014). 25456348 10.1021/am507391d

[b25] Yin H. J. . Ag@Au core-shell dendrites: a stable, reusable and sensitive surface enhanced Raman scattering substrate. Sci. Rep. 5, 14502 (2015). 26412773 10.1038/srep14502 PMC4585979

[b26] Shin K. S., Kim J. H., Kim I. H. & Kim K. Novel fabrication and catalytic application of poly (ethylenimine)-stabilized gold-silver alloy nanoparticles. J. Nanopart. Res. 14, 486–494 (2012).

[b27] Chen H. Y. . Green route for microwave-assisted preparation of AuAg-alloy-decorated graphene hybrids with superior 4-NP reduction catalytic activity. Ind. Eng. Chem. Res. 53, 17976–17980 (2014).

[b28] Loganathan B. . Surface enhanced vibrational spectroscopy and first-principles study of L-cysteine adsorption on noble trimetallic Au/Pt@Rh Clusters. Phys. Chem. Chem. Phys. 17, 21268–21277 (2015). 25650352 10.1039/c4cp05170j

[b29] Shang C. S., Hong W., Wang J. & Wang E. Carbon supported trimetallic nickel-palladium-gold hollow nanoparticles with superior catalytic activity for methanol electrooxidation. J. Power Sources 285, 12–15 (2015).

[b30] Hepel M., Dela I., Hepel T., Luo J. & Zhong C. Novel dynamic effects in electrocatalysis of methanol oxidation on supported nanoporous TiO_2_ bimetallic nanocatalysts. J. Electrochim. Acta 52, 5529–5547 (2007).

[b31] Nutta M. O., Hecka K. N., Alvarezb P. & Wong M. S. Improved Pd-on-Au bimetallic nanoparticle catalysts for aqueous-Phase trichloroethene hydrodechlorination. Appl. Catal. B-Environ. 69, 115–125 (2006).

[b32] Tang S. C., Tang Y. F., Gao F., Liu Z. G. & Meng X. K. Ultrasonic electrodeposition of silver nanoparticles on dielectric silica spheres. Nanotechnology 18, 295607 (2007).

[b33] Li T. . Electrocatalytic properties of hollow coral-like platinum mesocrystals. ACS Appl. Mater. Interfaces 4, 6941–6947 (2012). 10.1021/am302103e 23176064

[b34] Bi Y. P. & Lu G. X. Iodide ions control galvanic replacement growth of uniform rhodium nanotubes at room temperature. Chem. Commun. 47, 6402–6404 (2008). 10.1039/b814335h 19048169

[b35] Chen J. Y. . Ptical properties of Pd-Ag and Pt-Ag nanoboxes synthesized via galvanic replacement reactions. Nano Lett. 5, 2058–2062 (2005). 16218737 10.1021/nl051652u

[b36] Ma Y. Y. . Au@Ag Core-shell nanocubes with finely tuned and well-controlled sizes, shell thicknesses, and optical properties. ACS Nano 4, 6725–6734 (2010). 20964400 10.1021/nn102237c PMC2997519

[b37] Li H. B. . Te-template approach to fabricating ternary TeCuPt alloy nanowires with enhanced catalytic performance towards oxygen reduction and methanol oxidation reactions. J. Mater. Chem. A 3, 5850–5858 (2015).

[b38] Teng X. W. . Formation of Pd/Au nanostructures from Pd nanowires via galvanic replacement reaction. J. Am. Chem. Soc. 130, 1093–1101 (2008). 18161978 10.1021/ja077303e

[b39] Viyannalage L. T., Liu Y. & Dimitrov N. Processing of nanoporous Ag layers by potential-controlled displacement of Cu. Langmuir 24, 8332–8337 (2008). 18613704 10.1021/la800569t

[b40] Tan Y. W. . High-density hotspots engineered by naturally piled-up subwavelength structures in three-dimensional copper butterfly wing scales for surface-enhanced Raman scattering detection. Adv. Funct. Mater. 22, 1578–1585 (2012).

[b41] Tan Y. W. . Morphological effects on surface-enhanced Raman scattering from silver butterfly wing scales synthesized via photoreduction. Langmuir. 27, 11742–11746 (2011). 21875144 10.1021/la202445p

[b42] Tang S. C. . Highly catalytic spherical carbon nanocomposites allowing tunable activity via controllable Au-Pd doping. J. Colloid Interface Sci. 375, 125–133 (2012). 22425251 10.1016/j.jcis.2012.02.045

[b43] Deraedt C. . “Click” dendrimer-stabilized Pd nanoparticles as a green catalyst down to parts per million for efficient C-C cross-coupling reactions and reduction of 4-nitrophenol. Adv. Synth. Catal. 356, 2525–2538 (2014).

[b44] Vongehr S. . Adapting nanotech research as nano-micro hybrids approach biological complexity. J. Mater. Sci. Technol. 32, 387–401 (2016).

[b45] He Y. Q. . Fabrication of Au-Pd nanoparticles/graphene oxide and their excellent catalytic performance. Mater. Res. Bull. 51, 397–401 (2014).

[b46] Huang J. F. . Ag dendrite-based Au/Ag bimetallic nanostructures with strongly enhanced catalytic activity. Langmuir 25, 11890–11896 (2009). 19788231 10.1021/la9015383

[b47] Huang J. F. . Highly catalytic Pd-Ag bimetallic dendrites. J. Phys. Chem. C 114, 15005–15010 (2010).

[b48] Zhou L. . Site-specific growth of AgPd nanodendrites on Au bipyramids with remarkable catalytic performance. Nanoscale 6, 12971–12980 (2014). 25232660 10.1039/c4nr04190a

[b49] Hayakawa K., Yoshimura T. & Esumi K. Preparation of Gold-dendrimer nanocomposites by laser irradiation and their catalytic reduction of 4-nitrophenol. Langmuir 19, 5517–5521 (2003).

[b50] Kishore S. . Hydrogen storage in spherical and platelet palladium nanoparticles. J. Alloys Compd. 389, 234–242 (2005).

[b51] Corma A. & Pedro S. Chemoselective hydrogenation of nitro compounds with supported Gold catalysts. Science 313, 332–334 (2006). 16857934 10.1126/science.1128383

[b52] Zhang S. H. . Uniform Ni/SiO_2_@Au magnetic hollow microspheres: rational design and excellent catalytic performance in 4-nitrophenol reduction. Nanoscale 6, 7025–7032 (2014). 24841736 10.1039/c4nr00338a

[b53] Wang Y. G. . Concentration-dependent morphology control of Pt-coated-Ag nanowires and effects of bimetallic interfaces on catalytic activity. J. Mater. Sci. Technol. 32, 41–47 (2016).

[b54] An Q. . Fe_3_O_4_@carbon microsphere supported Ag-Au nanocrystals with the enhanced catalytic activity and selectivity for the reduction of nitroaromatic compounds. J. Phys. Chem. C 116, 22432–22440 (2012).

[b55] Ma A. . Interfacial nanodroplets guided construction of hierarchical Au, Au-Pt, and Au-Pd particles as excellent catalysts. Sci. Rep. 4, 4849 (2014). 24797697 10.1038/srep04849 PMC4010925

[b56] Harish S. . Synthesis of conducting polymer supported Pd nanoparticles in aqueous medium and catalytic activity towards 4-nitrophenol reduction. Catal. Lett. 128, 197–202 (2009).

