# Supplementary material for: Scalable Synthesis of Ag Networks with Optimized Sub-monolayer Au-Pd Nanoparticle Covering for Highly Enhanced SERS Detection and Catalysis
Source: Sci Rep. 2016 Nov 15;6:37092. doi: 10.1038/srep37092 (PMC5109471; doi:10.1038/srep37092)
Supplement: Supplementary Information [file srep37092-s1.doc]

**Supporting Information**

**Scalable Synthesis of Ag Networks with Optimized Sub-monolayer Au-Pd Nanoparticle Covering for Highly Enhanced SERS Detection and Catalysis**

**Tianyu Li,**^[a]^ **Sascha Vongehr,**^[a]^ **Shaochun Tang,*** ^[a]^ **Yuming Dai,**^[b]^ **Xiao Huang,**^[a]^ **Xiangkang Meng***^[a]^

^[a]^ *National Laboratory of Solid State Microstructures, College of Engineering Applied Sciences and Institute of Materials Engineering, Nanjing University, Jiangsu, P. R. China*

^[b]^ *School of Materials Engineering, Nanjing Institute of Technology, Jiangsu, P. R. China*

* Correspondence and requests for materials should be addressed to S.C. Tang (tangsc@nju.edu.cn) or X.K. Meng ([mengxk@nju.edu.cn](mailto:mengxk@nju.edu.cn)).

**1. Experimental Section**

**2. Theoretical calculations**

**Table S1.** HAuCl_4_ and K_2_PdCl_4_ concentrations and Ag precursor amounts used for the GRR synthesis, and the corresponding trimetallic Ag-Au-Pd networks’ formulas.

**Figure S1.** SEM images of Ag-Au-Pd networks obtained at different reaction temperatures of (a) room temperature, (b) 70 ^o^C, and (c-d) at high concentrations *C*_Au_ and *C*_Pd_ being 18.5 and 4.65 mM, respectively.

**Figure S2.** Plots of lnA_400nm_ versus reduction time *t* for the conversion from 4-NP to 4-AP with different tri-metallic Ag-Au-Pd networks obtained with a fixed Au/Pd ratio = 3.6 but using different weights of Ag precursor in the same amount of GRR solution.

**1. Experimental Section**

**1.1 Chemicals and Materials**

Silver nitrate (AgNO_3_), chlorauric acid (HAuCl_4_), Potassium tetrachloropalladate(II) (K_2_PdCl_4_), sodium chloride (NaCl), 4-nitrophenol (4-NP), and sodium borohydride (NaBH_4_) were purchased from Nanjing chemical Reagent No. 1 Factory. Glycerol and Ethylene glycol were obtained from Chemical Reagent Co., Ltd. of the National Pharmaceutical Group of China. All chemical reagents were analytical grade and used without further purification. A thermostatic oscillator was bought from Shanghai double shun industry development Co., Ltd. A JL-RO100Millipore-Q Plus water purifier supplied deionized water with a resistivity above 18.0 MΩ cm.

**1.2 Synthesis of Ag Nanosponges**

Ag nanosponges were first synthesized via fast reduction of metal salts followed by a slow network assembly in shaken ethanol-glycerol mixed solutions. Typically, 34 mg of AgNO_3_ was added to 4 mL distilled water with 6 mL glycerol in an ice-water bath, obtaining a clear, colorless 20 mM AgNO_3_ solution. During vigorous stirring, 3 mL hydrazine (85 wt%) was added quickly. Stirring in the ice-water bath continued for another 5 min, during which time the mixture turned opaque and gray. Then, under continuous stirring, 7 mL of ethanol was added into the mixed solution. The resulting suspension was subsequently transferred to the thermostatic oscillator with a constant temperature of 60 ^o^C. The oscillator shook the mixture at 80 r min^-1^. Gradually, the color of the suspension turned transparent again while a yellowish gray spongy solid gathered to float on the reaction medium after about 2 h. The shaking continued for another 30 min until the solution was colorless. The obtained Ag sponges look cotton-like yellowish gray and float on the remnants of the reaction solution. The float was washed for five times by distilled water, and finally dried in a freeze drying oven.

**1.3 Synthesis of Trimetallic AgAuPd Networks**

In a typical synthesis, 5.0 mg of the dried Ag nanosponges were immersed in 2 ml of mixed solutions containing typically 1.85 mM HAuCl_4_ and 0.46 mM K_2_PdCl_4_, as well as ethylene glycol. The volume ratio of water to ethylene glycol is 1:8. Continuous stirring was used to obtain a homogeneous suspension of the sponges.

Different HAuCl_4_ and K_2_PdCl_4_ concentrations were used for the GRR synthesis of tri-metallic Ag-Au-Pd networks. The replacement process was maintained for 10 minutes at 333.15 K using a water bath with a fast stir. The solution becomes colorless, and AgCl byproduct that may settle on the synthesized structures was removed with a saturated NaCl solution. Finally the resulting products were dried for 6 h at 40 ^o^C.

**1.4 Characterizations**

X-ray diffraction (XRD) measurements were conducted on a Shimadzu XRD-6000 instrument with Cu K*α* radiation (λ = 1.5418 Å). Scanning electron microscopy (SEM) was performed on a field-emission SEM microscope (Hitachi S-4800) operated at 5 kV. Transmission electron microscopy (TEM) analysis was conducted with a JEOL microscope (JEM-200CX) operated at 200 kV. The quantity of the individual elements contained in the products was analyzed by energy dispersed X-ray spectroscopy (EDS) and inductively coupled plasma-atomic emission spectroscopy (ICP-AES). EDS and elemental mapping analysis were carried out on the same SEM microscope; the ICP-AES measurement was performed on a J-A1100 ICP spectrometer (Jarrell-Ash Company, USA). To obtain accurate values, we performed the EDS analysis via collecting the information from a large area in a low-magnification SEM mode. Repeated measurements of compositional ratios in the SEM-EDS analyses were subsequently averaged. The valence states of elements were examined by X-ray photoelectron spectroscopy (XPS) performed in a Thermo VG Scientic MultiLab ESCA2000 system with a CLAM4 hemispherical analyzer and at a base pressure below 3×10^-10^ mbar. Photoelectrons were collected through the analyzer with pass energy of 20 eV.

- 1. **Catalysis and SERS Measurements**

To test the catalytic activity, 0.5 mg of dried trimetallic networks were added in 2.8 mL of 4-NP (7.0 × 10^-5^ M) under constant stirring at r.t. A freshly prepared aqueous solution of NaBH_4_ (0.20 mL, 0.1 M) was then added. The mixture was immediately transferred into a quartz cuvette with an optical path length of 1 cm. When the dried networks were put into the reaction solutions, they were suspended in the solution. However, as the catalytic reaction is being carried, such catalysts will deposit slowly at the bottom of the cuvette. To monitor changes in the reaction mixture, UV-visible absorption spectra were recorded with a Shimadzu UV-3600 UV-vis-NIR spectrophotometer. At such low concentration of catalyst, here ca. 0.17 g/L, the absorption by the Ag nanostructures’ surface Plasmon centered at around 400 nm can be disregarded. The UV-visible absorption spectrum of the aqueous mixture of 4-NP and NaBH_4_ has an absorption maximum at 400 nm due to the 4-NP ions under alkaline conditions. Time-dependent UV-visible absorption spectra show a drop in the absorption peak at 400 nm and concomitant development of a new peak at nearly 300 nm manifesting the production of 4-aminophenol from 4-NP. Due to the high concentration of BH_4_^-^ in the system, the decrease in BH_4_^-^ concentration is negligible during the process, and 4-AP is protected from oxidization due to air. The catalytic rate can therefore be evaluated by pseudo-first-order kinetics.

For SERS detection, 2 mL of Rhodamine B with a concentration of a 10^-6^ M was dropped on Ag nanosponges or trimetallic Ag-Au-Pd networks. After drying in air, SERS measurements were recorded at room temperature with a JY HR800 laser Raman spectrometer at an excitation wavelength of 532 nm (HeNe laser).

**2. Theoretical Calculations**

**2.1 From Desired Surface Coverage to *x***

The volume *V* and accessible area *A* of the ligaments are approximated by considering cylinders with radius *R*, therefore *V*/*A* = *R*/2. A number *N* of the NPs, each of radius *r*, volume *v*, and cross-sectional area *a*, together occupy an area (*N* *a*) that depends on how flat the particles are. The relation for half-spheres is *v*/*a* = 2*r*/3. The surface coverage ratio *σ* = (*N a*)/*A* of these particles on the ligaments is therefore approximately $\sigma=\frac{3R}{4r}s$ (1)

The factor *s* = (*N v*)/*V* is the volume added by the GRR divided by the original Ag volume, and since the atomic densities of Ag and Au are almost the same, it is also the ratio of the number of added atoms (denoted *Y*) over the number of initial Ag atoms (denoted *M*), therefore *s* = *Y*/*M.* To obtain Ag*_x_*Au*_y_*Pd*_z_*, for example at *y*/*z* = 3.6, the GRR removes *p* Ag atoms for every atom added, where *p* = (3*y* + 4*z*)/(*y* + *z*) = 3.2 (according to reaction equations 1 and 2 in the main text). For simplicity, we use *p* = 3, as if all new atoms are Au atoms. Taking differences between Pd and Au into account leads to negligible differences in the results. The atomic densities in bulk Ag and Au are about the same. Therefore, the GRR ratio of replaced volume (*p* *N v*) over original volume *V* is therefore *p* × *s* and proportional to *σ*. Thus, the amount of Ag left is

(*V* – 3*Nv*) [, which equals (*M* – 3 *Y*)], and so the resulting compound has the formula

$Ag_{x=(1-3s)/(1-2s)}(Au/Pd)_{1-x}$ (2)

[Below we also use *x* = (*M*-3*Y*)/(*M*-2*Y*)]. The NPs were initially expected to have a radius on the order of *r* ~ 5 nm, and the ligaments have a radius of about *R* ~ 45 nm. Aiming for half a monolayer (σ ~ 0.5), our equations predict *x* = 0.9, and we adjusted the concentrations therefore so as to obtain these atomic ratios.

**2.2 Relating Masses of Initial Ag to Molar Ratios**

*M* and *Y* are defined as above, namely, they are the number of initial Ag atoms and the number of added atoms, respectively. By *T* denote the total resulting number of atoms after the GRR. This leads to *Y* = (*y* + *z*) × *T.* For every atom added, *p* Ag atoms are removed. Therefore, *T* = *M* + (1 – *p*) *Y*. Applying *T* = *Y/*(*y+z*) leads to

$Y=(y+z)M+(y+z)(1-p)Y \Rightarrow Y=(y+z)M/[1+(y+z)(p-1)]$ (3)

The typical experiment used an *M* proportional to 5 mg and resulted in a certain *Y*. The same amount of GRR solution was used also in the new experiments of the second optimization step. Thus, also in these new experiments do the 5 mg lead to *x* = 0.9 and to the same *Y*. *Y* is the number of added atoms and thus constant in the new experiments. Thus, for the new (*y* + *z*) and *M* of the second optimization, called (*y*’ + *z*’) and *M*’, the following formulas hold: Firstly, *Y* = (*y*’+*z*’) *M*’/[1 + (*y*’+*z*’) (*p* – 1)], and since *Y* stays constant (“*Y* = *Y*’ ”), it holds

(*y*’+*z*’) *M*’ [1 + (*y+z*) (*p* – 1)] = (*y+z*) *M* [1 + (*y*’+*z*’) (*p* – 1)].

From this relation, the new (*y*’+*z*’) and thus *x*’ = 1 – (*y*’+*z*’) can be obtained by rearranging: $(y’+z’)=(y+z)M/M’/[1+(y+z)(p-1)(1-M/M’)]$ (4)

**Table S1.** Typical HAuCl_4_ and K_2_PdCl_4_ concentrations and Ag precursor amounts used for the GRR synthesis and the corresponding trimetallic Ag-Au-Pd networks’ formulas.

| Ag quantity  / mg | *C*_Au_/  (mM) | *C*_Pd_/  (mM) | Formula Ag*_x_*Au*_y_*Pd*_z_* | *y/z* |
| --- | --- | --- | --- | --- |
| 5.0 | 1.16 | 1.16 | Ag_0.92_Au_0.04_Pd_0.04_ | 1.22 |
| 5.0 | 1.39 | 0.93 | Ag_0.91_Au_0.06_Pd_0.03_ | 1.72 |
| 5.0 | 1.85 | 0.46 | Ag_0.90_Au_0.08_Pd_0.02_ | 3.55 |
| 5.0 | 1.98 | 0.33 | Ag_0.89_Au_0.09_Pd_0.02_ | 5.06 |
| 5.0 | 2.06 | 0.26 | Ag_0.89_Au_0.095_Pd_0.015_ | 5.53 |
| 4.0 | 1.85 | 0.46 | Ag_0.868_Ag_0.103_Pd_0.029_ | 3.55 |
| 4.5 | 1.85 | 0.46 | Ag_0.885_Ag_0.089_Pd_0.025_ | 3.55 |
| 5.5 | 1.85 | 0.46 | Ag_0.910_Ag_0.070_Ag_0.020_ | 3.55 |
| 6.0 | 1.85 | 0.46 | Ag_0.919_Ag_0.063_Ag_0.018_ | 3.55 |


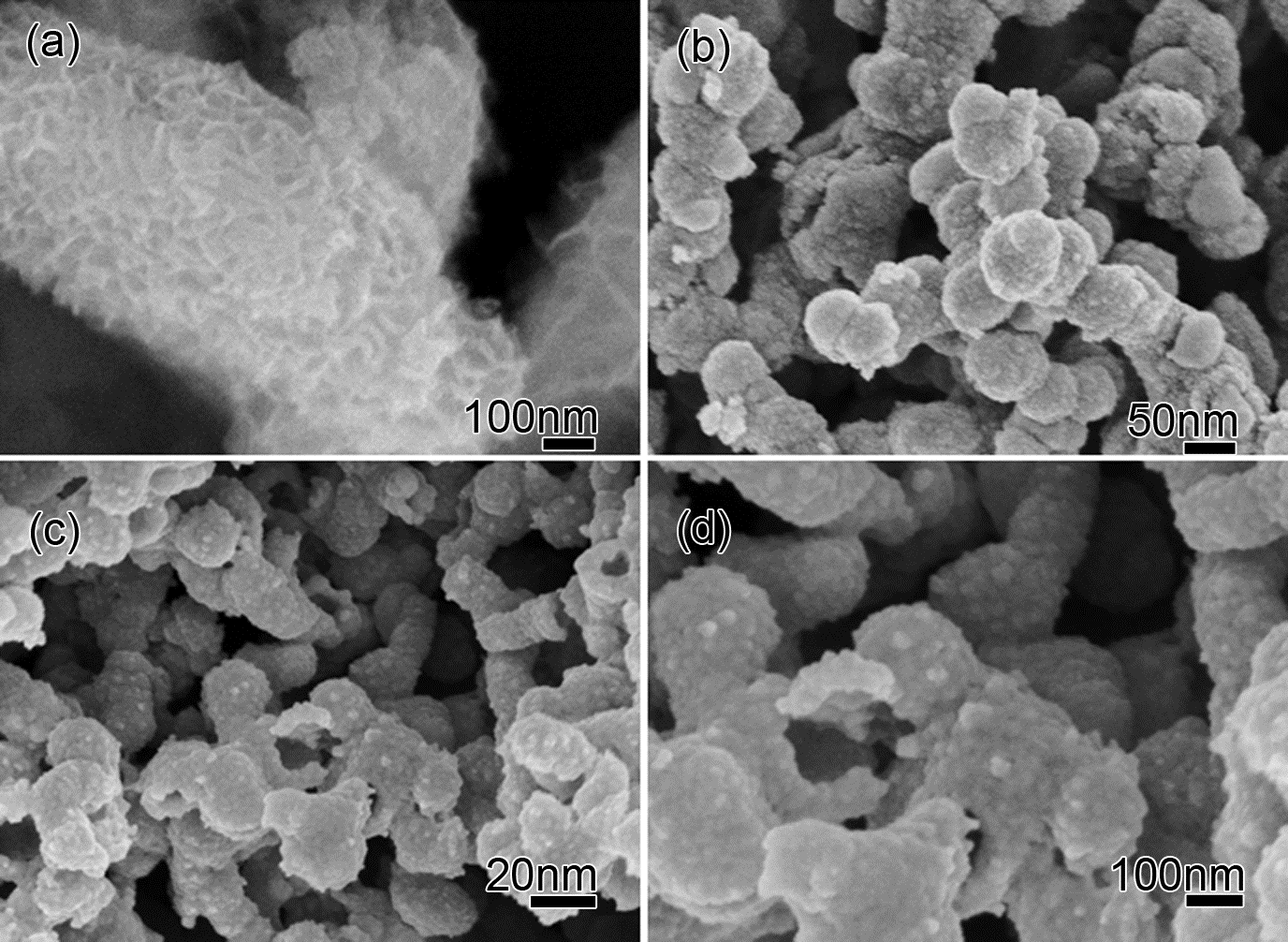


**Figure S1.** SEM images of Ag-Au-Pd networks obtained at different reaction temperatures of (a) room temperature, (b) 70 ^o^C, and (c-d) at high concentrations *C*_Au_ and *C*_Pd_ being 18.5 and 4.65 mM, respectively.


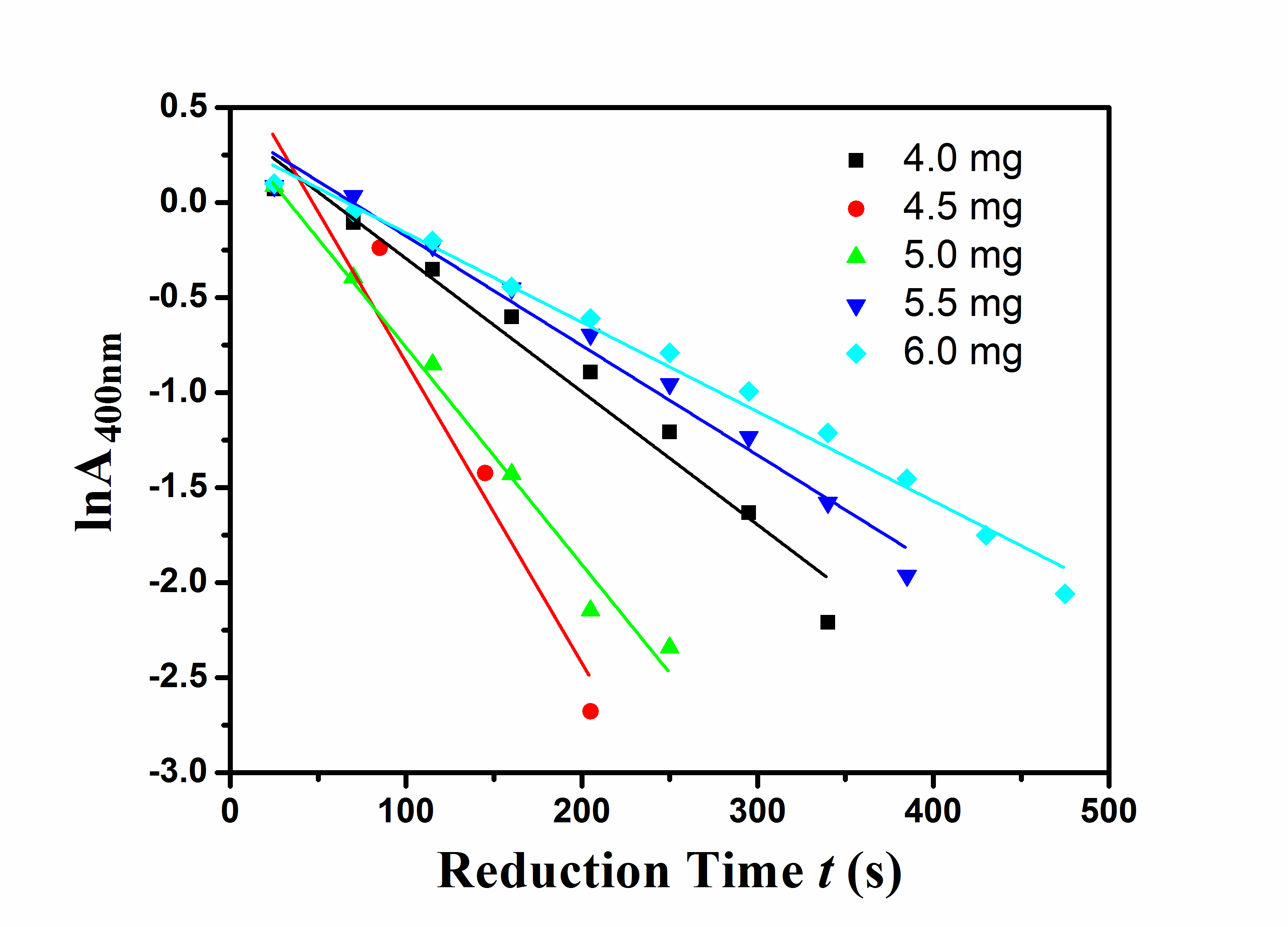


**Figure S2.** Plots of lnA_400nm_ versus reduction time *t* for the conversion from 4-NP to 4-AP with different tri-metallic Ag-Au-Pd networks obtained with a fixed Au/Pd ratio = 3.6 but using different weights of Ag precursor in the same amount of GRR solution.
